# Concentration-Dependent Solar Thermochemical CO_2_/H_2_O Splitting Performance by Vanadia–Ceria Multiphase Metal Oxide Systems

**DOI:** 10.34133/2020/3049534

**Published:** 2020-01-29

**Authors:** Asim Riaz, Muhammad Umair Ali, T. Gabriel Enge, Takuya Tsuzuki, Adrian Lowe, Wojciech Lipiński

**Affiliations:** ^1^Research School of Electrical, Energy and Materials Engineering, The Australian National University, Canberra ACT 2601, Australia; ^2^Department of Materials Science and Engineering, College of Engineering, Peking University, Beijing 100871, China; ^3^Research School of Earth Sciences, The Australian National University, Canberra ACT 2601, Australia

## Abstract

The effects of V and Ce concentrations (each varying in the 0–100% range) in vanadia–ceria multiphase systems are investigated for synthesis gas production *via* thermochemical redox cycles of CO_2_ and H_2_O splitting coupled to methane partial oxidation reactions. The oxidation of prepared oxygen carriers is performed by separate and sequential CO_2_ and H_2_O splitting reactions. Structural and chemical analyses of the mixed-metal oxides revealed important information about the Ce and V interactions affecting their crystal phases and redox characteristics. Pure CeO_2_ and pure V_2_O_5_ are found to offer the lowest and highest oxygen exchange capacities and syngas production performance, respectively. The mixed-oxide systems provide a balanced performance: their oxygen exchange capacity is up to 5 times higher than that of pure CeO_2_ while decreasing the extent of methane cracking. The addition of 25% V to CeO_2_ results in an optimum mixture of CeO_2_ and CeVO_4_ for enhanced CO_2_ and H_2_O splitting. At higher V concentrations, cyclic carbide formation and oxidation result in a syngas yield higher than that for pure CeO_2_.

## 1. Introduction

Oxygen-carrier-mediated solar thermochemical reduction–oxidation (redox) cycling is a promising approach to produce renewable fuels utilizing solar energy [1–8]. A typical solar-thermochemical redox cycle consists of two steps: (1) reduction of the oxygen carrier under an inert or reducing gas atmosphere (Reaction (1)) and (2) reoxidation of the reduced oxygen carrier by CO_2_ (Reaction (2)) and H_2_O (Reaction (3)). For methane as the reducing gas, the two-step cycle read:

(1) $$\begin{array}{c} \text{Methane partial oxidation }\left(\text{MPO}\right)\text{: }{\text{MO}}_{x}+\delta {\text{CH}}_{4}⟶{\text{MO}}_{x‐\delta }+\delta {\text{CO}}_{2}+2\delta {\text{H}}_{2} \end{array}$$

(2) $$\begin{array}{c} {\text{CO}}_{2}\text{ splitting }\left(\text{CDS}\right)\text{: }{\text{MO}}_{x‐\delta }+\delta {\text{CO}}_{2}⟶{\text{MO}}_{x}+\delta \text{CO} \end{array}$$

(3) $$\begin{array}{c} {\text{H}}_{2}\text{O splitting }\left(\text{WS}\right)\text{: }{\text{MO}}_{x‐\delta }+\delta {\text{H}}_{2}\text{O}⟶{\text{MO}}_{x}+\delta {\text{H}}_{2} \end{array}$$

The process results in the production of synthesis gas (syngas), a mixture of H_2_ and CO, which can be converted into liquid hydrocarbon fuels *via* the Fischer–Tropsch (FT) process [9–11]. In order to achieve high fuel (syngas) yields and high process efficiency in the redox cycling, the oxygen carriers should have a high oxygen exchange capacity and excellent cyclability [12]. Intensive research has been carried out to develop suitable catalyst/oxygen carriers. Some redox-material pairs such as Fe/FeO, CeO_2_/CeO_2-*δ*_, Mn_3_O_4_/MnO, and perovskites have been studied in the temperature range of 600–1500°C [9, 13–19]. The use of these materials has been demonstrated at laboratory and pilot-scale systems. However, their performance still requires improvements for the successful commercial deployment [20].

Ceria (CeO_2_) is one of the most efficient oxygen carriers with excellent oxygen ion mobility and redox kinetics. It shows high and stable fuel production rates *via* a nonstoichiometric oxygen exchange process. The fast redox kinetics and high fuel selectivity are distinct characteristics of CeO_2_ as compared to other redox materials [10, 11, 15, 21–27]. Fuel selectivity indicates the percentage of product gases (CO, H_2_) produced upon splitting of CO_2_, H_2_O, CH_4_, etc. Doping CeO_2_ with transition metals has been proposed to further improve the material chemical, thermal, mechanical, and optical characteristics. However, transition metal-doped CeO_2_ has so far demonstrated inferior redox reaction rates and oxygen ion mobility as compared to pure CeO_2_ [11, 26–28]. In addition, doped CeO_2_ shows extensive sintering at high temperatures, which decreases gas-phase mass transfer and consequently decreases the overall fuel production performance [10]. Hence, further research is required to develop new combinations of CeO_2_ and suitable cations to overcome the above-mentioned challenges.

Vanadia (V_2_O_5_) is recognized as one of the most efficient catalytic metal oxides. It is utilized for selective redox reactions in batteries [29] and gas-sensing applications [30, 31]. V_2_O_5_ supported with SiO_2_, TiO_2_, Al_2_O_3_, MgO, and CeO_2_ is also used in the selective oxidation of hydrocarbons for H_2_ and CO production [30–35]. Consequently, vanadia–ceria tertiary metal oxide systems have been widely studied for their structural, chemical, and oxidative properties [36]. Thermochemical redox performance and structural changes of vanadia–ceria multiphase systems were investigated for a sample with a V-to-Ce ratio of 25% [37]. However, the effects of V and Ce concentrations in vanadia–ceria systems have not been systematically studied.

In this work, we investigate the thermochemical performance of vanadia–ceria systems as oxygen carriers *via* solar thermochemical redox cycles for syngas production. Ultrafine particles of vanadia–ceria systems are produced using a facile liquid-phase precursor thermal-combustion method. V-to-Ce atomic ratios of 0 to 100% are investigated. The changes in the physicochemical characteristics of the oxide systems before and after methane looping reforming are studied. Sequential CO_2_ splitting (CDS) and water splitting (WS) reactions after methane partial oxidation (MPO) are investigated for syngas yield and purity. This study advances the field of solar thermochemistry towards achieving efficient and low-cost oxygen carriers for enhanced solar fuel production via thermochemical redox cycles.

## 2. Materials and Methods

### 2.1. Synthesis of Vanadia–Ceria Metal Oxide Systems

Ultrafine particles of pure CeO_2_, pure V_2_O_5_, and vanadia–ceria systems (V-to-Ce atomic ratios of 25%, 50%, and 75%, denoted as CV25, CV50, and CV75, respectively) are prepared using a liquid-phase precursor thermal-combustion method [38]. This technique allows for a large-scale production of the nanoparticles with controlled particle size and morphology. Briefly, stoichiometric ratios of Ce (III) nitrate hexahydrate (CeN_3_O_9_·6H_2_O, Aldrich) and vanadium oxytripropoxide (VC_9_H_21_O_4_, Aldrich) precursors are dissolved separately in a mixture of 4 mL ethanol and 4 mL deionized (DI) water, respectively. After stirring for 1 hour, both solutions are mixed together and stirred for 3 hours at room temperature. Finally, the precursor solutions are transferred into an alumina crucible and heat treated at 1173 K for 3 hours. During the heat treatment, temperature is raised stepwise: first, the temperature is held at 353 K for 1 hour, then increased to 1173 K at a ramp rate of 3 K min^−1^.

### 2.2. Thermochemical Cycling

The cyclic thermochemical redox performance of metal oxide powders is evaluated in a vertical tube reactor (99.98% Al_2_O_3_) placed inside an infrared gold image furnace (P4C-VHT, Advance Riko). Highly porous and refractory alumina fiber mats (AIBF-1, 97% Al_2_O_3_ and 3% SiO_2_, ZIRCAR) are used as a sample stage and as an upper protective layer for the powder samples (250 ± 10 mg). To facilitate the solid–gas transfer, a gap of ~2 cm is set between the powder and the upper protective layer mat. The samples and fiber layers are placed in the tube axially in the middle of heating zone to ensure uniform heating. The LabVIEW (National Instruments) software platform is used to operate the flow rate controllers (F201CV, Bronkhorst) and actuated valves (1315R, Swagelok) to achieve desirable gas flow rates in “mL min^−1^.” A B-type thermocouple sealed in an alumina sheath is located immediately beneath the fiber mat sample stage to monitor the sample temperature. The composition of reactant and product gasses is recorded by a quadrupole mass spectrometer (OmniStar™ GSD 320, Pfeiffer Vacuum). A schematic of the experimental setup used for thermochemical redox cycling is shown in Figure [Supplementary-material supplementary-material-1].

First, Ar (grade 5.0) gas is purged with a flow rate of 500 mL min^−1^ to remove any gas species (H_2_, CO, and CO_2_) from the surface of the tube and gas lines. The reactor is heated under an Ar flow (500 mL min^−1^) from ambient temperature to an optimized isothermal operating temperature of 1173 K at a ramp rate of 100 K min^−1^. The reduction (methane partial oxidation, MPO) of the powder samples is carried out under an 8% CH_4_ flow (20 mL min^−1^, grade 4.5) diluted with 92% of Ar (230 mL min^−1^). The reduced samples are reoxidized by a 4% CO_2_ flow (10 mL min^−1^) during CO_2_ splitting (CDS) reactions. For H_2_O splitting (WS) reactions, steam vapor is generated at 368 K in a water bubbler filled with DI water, then an Ar flow of 30 mL min^−1^ is passed through the bubbler to carry H_2_O vapors and further diluted with an Ar flow of 220 mL min^−1^ before it is delivered to the tubular reactor. Inert gas sweeping with Ar is done with a flow rate of 500 mL min^−1^ before and after each reduction and oxidation step of the thermochemical cycle. Four different redox sequences are investigated and denoted as MPO–CDS, MPO–WS, MPO–WS–CDS, and MPO–CDS–WS. The details of the sequence and duration of each step and gas flow rates during these redox cycles are as follows:

(4) $$\begin{array}{c} \text{MPO}–\text{CDS}:\text{Ar}\left(5\;\min\right)/{\text{CH}}_{4}\left(20\;\min\right)/\text{Ar }\left(5\;\min\right)/{\text{CO}}_{2}\left(10\;\min\right)\\ \text{MPO}–\text{WS}:\text{ Ar}\left(5\;\min\right)/{\text{CH}}_{4}\left(20\;\min\right)/\text{Ar}\left(5\;\min\right)/{\text{H}}_{2}\text{O}\left(10\;\min\right)\\ \text{MPO}–\text{WS}–\text{CDS}:\text{Ar }\left(5\;\min\right)/{\text{CH}}_{4}\left(20\;\min\right)/\text{Ar}\left(5\;\min\right)/{\text{H}}_{2}\text{O}\left(7\;\min\right)/\text{Ar}\left(5\;\min\right)/{\text{CO}}_{2}\left(7\;\min\right)\\ \text{MPO}–\text{CDS}–\text{WS}:\text{Ar }\left(5\;\min\right)/{\text{CH}}_{4}\left(20\;\min\right)/\text{Ar}\left(5\;\min\right)/{\text{CO}}_{2}\left(7\;\min\right)/\text{Ar}\left(5\;\min\right)/{\text{H}}_{2}\text{O}\left(7\;\min\right) \end{array}$$

The redox performance of ultrafine powders is evaluated over 10 cycles. The vanadia–ceria systems are structurally and chemically analyzed before and after 10 cycles, and the results are compared to those obtained with the as-prepared samples.

### 2.3. Material Characterization

All powder samples are characterized as prepared and after 10 thermochemical cycles. X-ray diffraction (XRD) analysis of powder samples is carried out using a D2 phaser diffractometer (Bruker) with a Cu k*α* (1.54 Å) radiation source operated at 300 W power (30 kV, 10 mA). XRD patterns are recorded in a diffraction angle range of 10–80° with a step width of 0.02° and a scanning rate of 0.75°min^−1^. The Scherrer equation is applied onto the most intense peaks of XRD patterns to calculate the crystallite size of the powders.

X-ray photoelectron spectroscopy (XPS) analysis is carried out using a ThermoFisher ESCALAB 250Xi X-ray photoelectron spectrometer equipped with a 180° double focusing hemispherical analyzer. A monochromatic Al k*α* source with a spot size of 200–900 *μ*m is utilized at 12 kV and 12 mA. A total pressure in the chamber is 10^−8^ mbar. Samples are scanned at various spots on an area of 25 mm^2^ and depth of 4 mm with a beam energy of 40–160 eV. XPS spectra are processed using the CasaXPS software version 2.3.18 (Casa software Ltd., Teignmouth, UK). The binding energy of aliphatic carbon peak C 1s at 284.8 eV is used as a reference in the survey spectra.

A Raman imaging microscope (Renishaw Plc, model 2000) equipped with an Olympus BH2 microscope is utilized for the structural analysis of powder samples. Samples are placed on a motorized XYZ stage of the microscope equipped with an air-cooled CCD detector and a CCD camera. The excitation wavelength of the NIR laser is 785 nm. Raman spectra are recorded in a Raman shift range of 100–1200 cm^−1^. The exposure time is 20 s with an accumulation up to 3, and laser power is adjusted in the range of 0.01–0.5% (<6 MW), depending on the sample response to laser excitation.

Morphological information of the powder before and after the redox cycles is obtained by using a field emission scanning electron microscope (FESEM, Zeiss Ultraplus). A high-resolution transmission electron microscope (HR-TEM, JEOL 2100F) is utilized for the measurements of particle size distribution and lattice spacing. Operating voltage of the microscope is adjusted up to 200 kV according to the resolution and sample response. A lacey carbon-coated 200 mesh copper grid is used as a substrate for samples. The powder is first dispersed in ethanol, and a drop of particle suspension is dried on the copper grid. Information about composition of samples is obtained by energy dispersive X-ray spectroscopy elemental mapping (EDX) analysis carried out by scanning transmission electron microscopy (STEM) mode on JEOL 2100F.

The quantification of Ce and V was carried out using an Agilent 5110 ICP-OES (Agilent Technologies, Australia), operating in Synchronous Vertical Dual View (SVDV) mode, allowing simultaneous detection of axial and radial emission signals. The sample introduction system was made up of a double pass cyclonic spray chamber, a SeaSpray nebuliser, and a 2.4 mm quartz injector. Operating parameters for ICP-OES analysis of oxygen carriers before and after cycling are tabulated in Table [Supplementary-material supplementary-material-1]. All dilutions and sample preparation for ICP-OES measurement were performed using ultrapure water (MilliQ, Merck), as well as subboiling distilled HNO_3_. Calibration solutions for Ce and V measurements were prepared from single element solutions in concentrations ranging from 0.1 to 10 *μ*g mL^−1^; for analysis, samples were diluted to fall within the calibration curve. Ce and V from a single element standard were diluted to 0.1, 0.5, 1, 5, and 10 ppm concentrations to make a calibration curve for each element.

## 3. Results and Discussion

**Figure 1:**
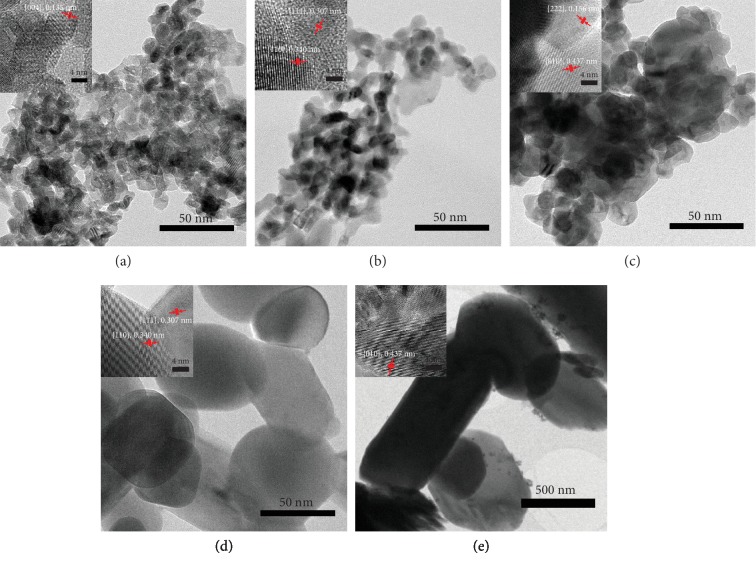
Morphology and size estimation: transmission electron microscopy images of as-prepared (a) pure CeO_2_, (b) CV25, (c) CV50, (d) CV75, and (e) pure V_2_O_5_. The inserts in each image shows the lattice spacing and crystal planes of the vanadia–ceria system.

Figure 1 shows transmission electron microscopy (TEM) images of as-prepared vanadia–ceria systems, at the same magnification. The particle size increases with increasing V concentration. The average particle size of pure CeO_2_ is 12 ± 3 nm, which increases to 55 ± 5 nm with addition of 75% V, while pure V_2_O_5_ has the highest average particle size of 89 ± 3 nm. Field emission scanning electron microscopy (FESEM) images of as-prepared vanadia–ceria systems confirm these values (Figure [Supplementary-material supplementary-material-1]).

The morphological study of vanadia–ceria systems after MPO–CDS cycles is carried out by FESEM. It reveals an extensive sintering in pure V_2_O_5_ and CeO_2_ samples, showing individual particles fused into large microparticles. However, the sintering is prominent in V-rich vanadia–ceria mixed-metal-oxide particles (Figure [Supplementary-material supplementary-material-1]). The TEM image of a reduced CV75 sample before MPO–CDS cycles shows sintering of small particles forming a sheet like morphology, while individual particles can also be observed (Figure [Supplementary-material supplementary-material-1]). The CV75 sample after MPO–CDS cycles shows extensive sintering which results in increased particle sizes of >500 nm (Figure [Supplementary-material supplementary-material-1]).

**Figure 2:**
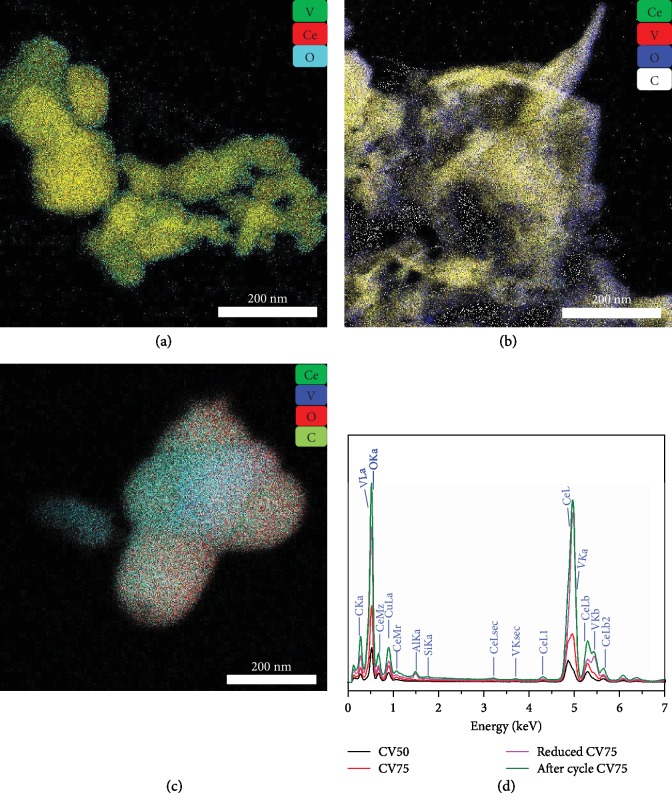
Elemental analysis of vanadia–ceria systems: overlay images of scanning transmission electron microscope elemental mapping of CV75 samples: (a) as-prepared, (b) reduced, and (c) after MPO–WS–CDS cycles, representing the distribution of Ce, V, C, and O; (d) energy dispersive spectra of the scanned area of CV50, CV75, reduced CV75, and cycled CV75 samples, showing the amounts of Ce, V, O, and C with respect to the binding energies of their respective orbitals.

Energy dispersive spectra (EDS) and an overlaid elemental map of as-synthesized as well as reduced CV75 samples before and after MPO–WS–CDS cycles are shown in Figure 2. As-prepared samples show no noticeable presence of carbon. Since V-K*α* and Ce-L peaks are situated in the same energy range in EDS spectra, the presence of both elements shows a combined overlay color (Figures 2(a)–2(c)). A nonuniform distribution of V in CV75 is observed after MPO–WS–CDS cycles, indicating a possible V loss during the redox cycles or segregation of V_2_O_5_ and CeO_2_ phases in powder samples.

**Figure 3:**
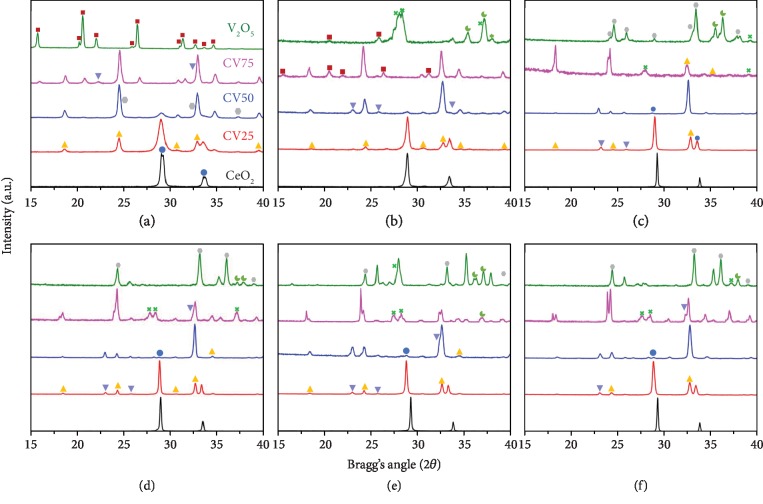
Structural analysis of vanadia–ceria systems: XRD patterns of (a) as-prepared and after cycled (b) reduced, (c) MPO–CDS, (d) MPO–WS, (e) MPO–WS–CDS, and (f) MPO–CDS–WS, pure ceria, CV25, CV50, CV75, and pure V_2_O_5_ representing the evolution of CeVO_4_ as a function of vanadium content and structural changes in after-cycling. Spheres, upward cones, hexagons, downward cones, squares, crosses, and crescents represent CeO_2_, CeVO_4_, V_2_O_3_, CeVO_3_, V_2_O_5_, VO_2_, and VC phases, respectively.

XRD patterns of as-prepared CeO_2_, V_2_O_5_, and vanadia–ceria ultrafine particles are presented in Figure 3(a). The diffraction patterns of pure CeO_2_ and V_2_O_5_ are in good agreement with that of cubic CeO_2_ (JCPDS # 72-0076) and orthorhombic V_2_O_5_ (JCPDS # 75-0298), respectively. Addition of V in CeO_2_ promotes the formation of cerium vanadate:*V*_2_*O*_5_ + 2*CeO*_2_⟶2*CeVO*_4_ + 1/2*O*_2_ (CeVO_4_, JCPDS # 72-0282), accompanied by a change in the valence of Ce from Ce^+4^ to Ce^+3^, while the valence of V remains V^5+^. With increasing V content to 25%, an increase in the CeVO_4_ phase with a considerable amount of CeO_2_ is observed. A maximum conversion of CeO_2_ into CeVO_4_ is achieved with 50% V loading, while a small amount of CeO_2_ remains in the structure. With further increase of V content to 75%, the amount of CeVO_4_ becomes the smallest (16.9%) with a major portion of V_2_O_3_, as listed in Table [Supplementary-material supplementary-material-1]. The structural changes caused by the addition of V greatly affect the rates of thermochemical redox reactions, which is discussed in the following sections.

XRD patterns of reduced vanadia–ceria systems are presented in Figure 3(b). Reduced pure CeO_2_ does not undergo any structural change except shifting of diffraction peaks to lower angles, possibly caused by oxygen depletion. However, reduction of pure V_2_O_5_ results in the conversion of V^5+^ to V^4+^, which can be seen as the presence of a VO_2_ phase in the XRD pattern of reduced V_2_O_5_ and CV75 [39]. The presence of vanadium carbide (JCPDS#89-1096) and metallic V indicates the catalytic interaction of pure V_2_O_5_ with methane to produce carbon during the methane partial oxidation reaction [40–42]. A decline in the peak intensities of a CeVO_4_ phase is observed for CV25, which indicates the conversion of V^5+^ to V^3+^ due to the formation of a CeVO_3_ phase.

The XRD patterns of pure V_2_O_5_, CV25, CV50, and CV75 samples after 10 consecutive cycles of MPO–CDS, MPO–WS, MPO–WS–CDS, and MPO–CDS–WS are presented in Figures 3(c)–3(f). In CV25, the diffraction angles of CeVO_4_ shift to lower angles after 10 MPO–CDS cycles. The presence of peaks associated with CeVO_3_ indicates the partial oxidation of CeVO_3_ to CeVO_4_ due possibly to incomplete recovery of oxygen. Segregated phases of CeVO_3_, CeVO_4_, and VO_2_ are observed in the XRD patterns of CV75. However, oxidation of reduced V_2_O_5_ results in the oxidation of vanadium carbide and metallic V to V_2_O_3_ and VO_2_. The XRD analysis of V_2_O_5_–CeO_2_ systems after the redox cycles reveals that the formation of a CeVO_4_ phase increases with increasing V content, reflecting contribution of V in the redox reactions. Ce^3+^ sites are stabilized by the valence change (Ce^4+^ to Ce^3+^) during the formation of CeVO_3_/CeVO_4_ [32, 36].

The surface analysis of as-prepared samples complements the findings of XRD analysis. A typical XPS spectrum of pure CeO_2_ is composed of two multiplets of 3d 3/2 at 900 and 898 eV, 3d 5/2 at 888 and 882 eV, and neighboring 2 peaks at 907 and 916 eV [43], as presented in Figure 3(a). The locations of these six peaks corresponding to the spin-orbit doublets of 3d 3/2 and 5/2 are in good agreement with the reported XPS analysis of Ce^4+^/Ce^3+^ [43]. The binding energy of O 1s is 529.02 eV, corroborating the presence of lattice oxygen species, while the neighboring peak at 531.46 eV refers to the presence of adsorbed oxygen molecules (Figure [Supplementary-material supplementary-material-1]). The binding energy of Ce 3d3/2 peaks changed from 888.5 eV to 885.2 eV with increasing V contents, due to an increase in CeVO_4_ content. In addition, the disappearance of Ce^4+^ peak at ~916 eV confirms the change of Ce valence from +4 to +3 due to increasing concentrations of CeVO_4_ in CV75 sample.

**Figure 4:**
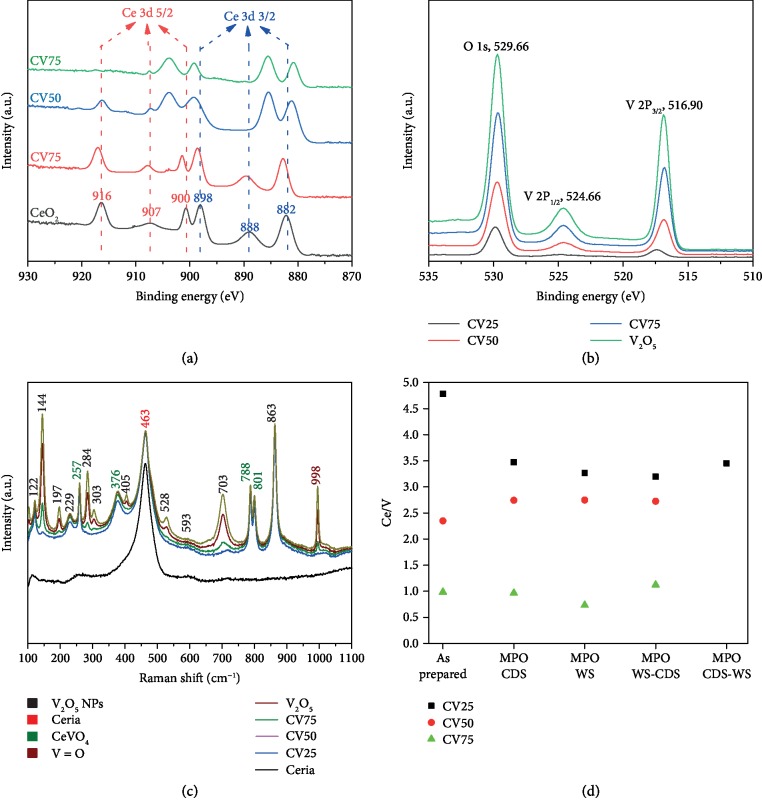
Chemical analysis of vanadia–ceria systems: XPS spectra of as-prepared vanadia–ceria metal oxide systems in the (a) Ce 3d and (b) V 2P and O 1s regions. (c) Raman spectra of as-prepared pure CeO_2_, CV25, CV50, CV75, and pure V_2_O_5_ samples, where peaks associated with the evolution of surface V_2_O_5_ and CeVO_4_ with increasing V content, are labeled with black and green-colored numbers, respectively. (d) Ce/V ratios in as-prepared and cycled CV25, CV50, and CV75 samples, obtained by ICP-OES analysis.

The binding energies of 516.9 eV and 524.66 eV shown in Figure 4(b) correspond to V 2p_3/2_ and V 2p_1/2_ spin orbits of pure V_2_O_5_, respectively, depicting the +5 valence state of V [44]. In addition, 3d 3/2 to 3d 5/2 ratio decreases with increasing V content in vanadia–ceria systems. An increase in the binding energies of O 1s with V addition also indicates the presence of Ce (III) states and Ce–O–V interactions.

XPS spectra of reduced V_2_O_5_, CV25, and CV75 are shown in Figure [Supplementary-material supplementary-material-1]. Higher V(V) content can be confirmed from the increasing intensities of V 2P_1/2_ and V 2P_3/2_ in Ce–V systems with V content (CV25⟶CV75⟶pure V_2_O_5_). The O 1s peaks shift to high binding energies with higher V content, due to higher CeVO_4_ contents [43]. An additional shoulder peak of V 2P_3/2_ is observed in reduced V_2_O_5_ due to the presence of VO_2_. An additional O 1s peak is also observed at 531.89 eV for reduced V_2_O_5_. After the first MPO–CDS cycle, the additional O 1s peak merged into the main O 1s peak. This may be due to neighboring V species with multiple oxygen states. High concentrations of surface-adsorbed oxygen molecules may also contribute to the presence of the additional O 1s peak.

The information obtained from the XPS spectra provides an insight into the phenomenon of possible V volatilization. By obtaining Ce/V and V/O ratios, the loss of V can be quantified. In as-prepared powders, the V/O ratio increases, and Ce/V ratio decreases with increasing the V content. After the methane partial oxidation reaction, an increase in the V/O and Ce/V ratios is observed due to oxygen and V loss. However, after 10 consecutive cycles, a further decrease of the V/O ratio is observed, which suggests the loss of V and incomplete oxygen recovery. Ce/V segregation is also a possible reason for variable V concentrations at the surface. This can be further investigated by quantifying Ce and V concentrations in the bulk *via* the ICP-OES technique with a precision of up to parts per billion (ppb) level. The Ce/V ratios before and after thermochemical redox cycles are presented in Figure 4(d). An expected decline in the Ce/V ratio is observed in as-prepared Ce–V oxide samples due to higher V content. However, an irregular trend is observed after redox cycles, which suggests that segregation of Ce/V and V loss both contribute to the variable concentrations of V.

Raman spectra of vanadia–ceria systems are presented in Figure 4(c). Pure CeO_2_ has a Raman shift at 461 cm^−1^ [30, 45], while pure V_2_O_5_ shows multiple signature peaks at 122, 144, 197, 229, 257, 284, 303, 376, 405, 463, 528, 703, 788, 801, and 863 cm^−1^ [46, 47]. The V=O interaction seen at 998 cm^−1^ is dominant in pure V_2_O_5_ and CV75, while it diminishes in CV25 and CV50 samples. The intensities of peaks at 144, 197, 284, 303, 405, 528, and 703 cm^−1^ indicate the presence of CeVO_4_.

### 3.1. Thermochemical Redox Performance

**Figure 5:**
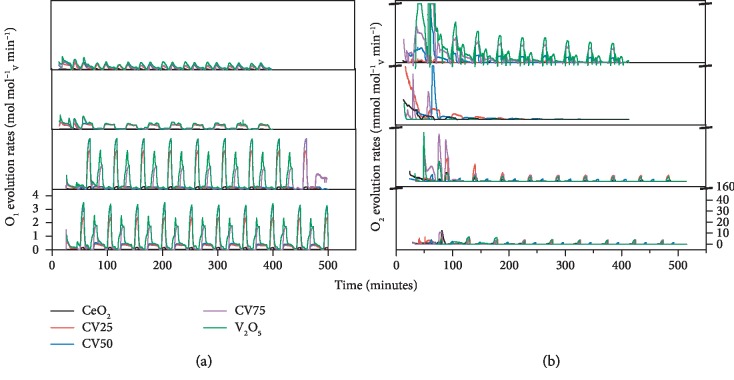
Oxygen exchange performance of vanadia–ceria metal oxide systems: (a) oxygen evolution rates calculated from the amounts of CO and CO_2_ (referred in text as O1) and (b) oxygen signal from a mass spectrometer (referred in the text as O2) during the reduction and oxidation steps of MPO–CDS, MPO–WS, MPO–WS–CDS, and MPO–CDS–WS redox cycles. The vertical-scale break in (b) denoted by the dash is from 50 to 150 mmol mol_V_^‐1^min^−1^.

The performance of vanadia–ceria systems is evaluated based on the oxygen exchange capacity and the yield of syngas per mole of V during 10 consecutive thermochemical redox reaction cycles. Moles of Ce ions are considered for the calculation of rates and yields of syngas production for pure CeO_2_ sample.  Figure 5 shows the oxygen evolution rates during reduction and oxidation steps of MPO–CDS, MPO–WS, MPO–WS–CDS, and MPO–CDS–WS cycles. The oxygen rates calculated from CO/CO_2_ evolution rates are referred to as “O1 rates,” while oxygen rates deduced directly from the oxygen signal obtained during gas analysis are referred to as “O2 rates” in the following discussion. This set of data provides an insight into material's ability to react with reducing and oxidizing atmospheres.

During MPO–CDS cycles, pure CeO_2_ shows stable average O1 evolution rates of around 0.08 mol mol^−1^_Ce_ min^−1^ with a peak of 0.107 mol mol^−1^_Ce_ min^−1^. In contrast, pure V_2_O_5_ exhibits the highest rates of 0.5 mol mol^−1^_V_ min^−1^. An increase in O1 rates from 0.27 mol mol^−1^_V_ min^−1^ to 0.43 mol mol^−1^_V_ min^−1^ is observed with addition of V from 25% to 75%. In addition, the vanadia–ceria multiphase system demonstrates high and stable oxygen evolution rates during the CO_2_ splitting reactions with the highest rate of 0.35 mol mol^−1^_V_ min^−1^ for 75%V.

During MPO–WS redox cycles, a similar trend of increasing O1 rates with the addition of V (25–75%) content is observed. Pure V_2_O_5_ shows the highest oxygen evolution rates up to 0.45 mol mol^−1^_V_ min^−1^, followed by CV75 with rates up to 0.38 mol mol^−1^_V_ min^−1^, during the reduction step of MPO–WS redox cycles. Interestingly, pure V_2_O_5_ shows considerable O1 rates in water splitting reaction, which refers to oxidation of vanadium-carbide carbon species formed during methane partial oxidation reaction, *C* + *H*_2_*O*⟶*CO* + *H*_2_. This phenomenon supports the findings of XRD analysis of reduced and oxidized pure V_2_O_5_.

Following this result, in order to investigate the effect of oxidation atmosphere on the reactivity of reduced oxygen carriers with steam and CO_2_, a combination of WS and CDS reactions following the methane partial oxidation step, i.e., MPO–WS–CDS, is performed. During the methane partial oxidation step of MPO–WS–CDS cycles, pure V_2_O_5_ and CV75 demonstrate the highest O1 rates of 3.75 mol mol^−1^_V_ min^−1^, closely followed by CV50 at 3.69 mol mol^−1^_V_ min^−1^ and CV25 at 2.85 mol mol^−1^_V_ min^−1^. During the WS step, the O1 rates are the highest for pure V_2_O_5_ followed by the rates for CV75, which confirms the findings of vanadium carbide presence in pure V_2_O_5_ and CV75. Lowering the V content minimizes the carbide formation, resulting in pure H_2_ release during water splitting reaction. Consequently, CV25 and CV50 show moderate to high O1 rates with pure H_2_ production during MPO–WS–CDS cycles.

A similar trend is observed during the MPO–CDS–WS cycles. During the methane partial oxidation reaction, pure V_2_O_5_ shows the highest O1 rates at 3.35 mol mol^−1^_V_ min^−1^, closely followed by CV75 and CV50 at 3.25 mol mol^−1^_V_ min^−1^. However, the O1 rates significantly increase during the oxidation step, with more than fivefold increase in CO production as compared to MPO–CDS cycles. The high VO_2_ content observed in the XRD patterns also confirms a higher oxygen recovery in reoxidized pure V_2_O_5_ and CV75 samples during MPO–CDS–WS cycles as compared to V_2_O_5_ and CV75 reoxidized to V_2_O_3_ during MPO–CDS cycles. An increase in the CeVO_4_ phase also supports efficient reoxidation of CV25 and CV50 during MPO–CDS–WS cycles.

The rates of O2 evolution during all four types of redox cycles are presented in Figure 5(b). The O2 oxygen rates increase with V content (25–100%) during the methane partial oxidation step of MPO–CDS cycles, while the highest O2 rate of 20 mmol mol^−1^_V_ min^−1^ is observed for pure V_2_O_5_. The evolution rates tend to decrease over multiple cycles due to the oxidation of the deposited carbon and the carbide formation. Interestingly, no considerable O2 rates are observed after the first MPO–WS cycle, while these rates are high in sequential WS and CDS cycles. In addition, O2 rates are higher for high-Ce systems than high-V systems. This is in agreement with the results of XRD study, where V^4+^ of VO_2_ is observed in CV75 and pure V_2_O_5_ samples, representing better reoxidation capacity of these samples. In contrast, V^3+^ of CeVO_3_ is observed in CV25 and CV50 samples, demonstrating incomplete reoxidation during WS reactions in these samples.

**Figure 6:**
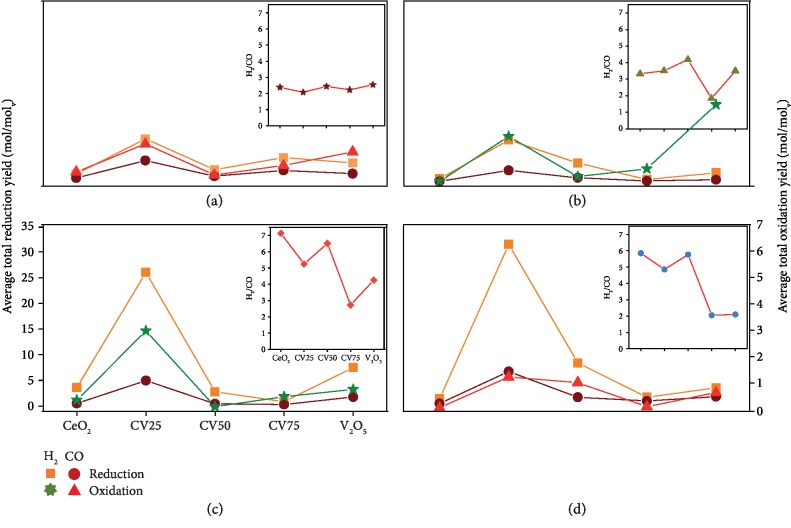
Performance evaluation of syngas production yield: average total yield of syngas (H_2_ and CO) produced during reduction and oxidation steps of 10 consecutive (a) MPO–CDS, (b) MPO–WS–CDS, (c) MPO–WS, and (d) MPO–CDS–WS redox cycles. The inserts represent the H_2_/CO ratio during different redox cycles.

The average total yield of syngas during the reduction and oxidation reactions over 10 redox cycles is presented in Figure 6. In MPO–CDS cycles, CV25 shows the highest H_2_ and CO yields, up to 8.2 mol mol^−1^_V_ and 3.95 mol mol^−1^_V_, respectively, and a H_2_/CO ratio of 2.21. This is followed by the H_2_ and CO yields observed for pure V_2_O_5_, 3.54 mol mol^−1^_V_, and 1.39 mol mol^−1^_V_, respectively. As discussed earlier, carbon deposition is observed during MPO–WS cycles, while higher V contents promote the cyclic oxidation of vanadium carbide. This phenomenon leads to a controlled H_2_/CO ratio with a minimum of 2.6 with 75% V, while all other samples show their H_2_/CO ratios greater than 4. A combination of WS and CDS cycles significantly improves the H_2_/CO ratio, where CV25 produces the highest syngas yield with a moderately high H_2_/CO ratio. During MPO–CDS–WS cycles, the H_2_/CO ratios tend to increase drastically in Ce-rich samples up to 50%V as compared to pure V_2_O_5_ and CV75. Despite the decline in performance, Ce-rich vanadia–ceria systems demonstrate stable reaction rates as compared to the rates obtained with CV75 and pure V_2_O_5_. The average total yields of H_2_ and CO for pure V_2_O_5_ are 3.8 mol mol^−1^_V_ and 1.04 mol mol^−1^_V_, respectively. The highest yields of H_2_ (31.24 mol mol^−1^_V_) and CO (6.56 mol mol^−1^_V_) are obtained for CV25, with a H_2_/CO ratio of 4.7.

The average total fuel yield during the oxidation step of MPO–CDS, MPO–WS, MPO–WS–CDS, and MPO–CDS–WS cycles is shown in Figure 6. During MPO–CDS cycles, CV25 showed the highest CO yield of 1.59 mol mol^−1^_V_, followed by pure V_2_O_5_ with 1.29 mol mol^−1^_V_. However, the fuel production rates for CV75 are more stable than those for pure V_2_O_5_, as discussed previously. The high yield of CO during oxidation of pure V_2_O_5_ also suggests the presence of carbon species deposited in V_2_O_5_. During MPO–WS cycles, no considerable CO is observed in Ce-rich vanadia–ceria systems containing up to 50% V, for which a high H_2_ yield of 3.04 mol mol^−1^_V_ is observed. Here, the H_2_ yield decreases with the increasing V content. Sequential WS and CDS cycles result in an improved H_2_/CO ratio and a high H_2_ yield during WS reaction. Pure V_2_O_5_ shows the highest average total H_2_ yield of 3.07 mol mol^−1^_V_, followed by 1.87 mol mol^−1^_V_ for CV25. Considerable amounts of H_2_ are observed during the WS step of MPO–CDS–WS cycles, indicating incomplete reoxidation of reduced samples by CO_2_. Furthermore, an addition of WS steps to MPO–CDS cycles lowers the CO yield during the CDS step, as compared to MPO–CDS cycles. By analyzing the product yields of the methane partial oxidation reaction during MPO–CDS–WS and MPO–CDS cycles, it is found that water splitting reaction suppresses methane reforming and promotes methane cracking, increasing the H_2_/CO ratio. The subsequent CDS reaction results in gasification of the deposited carbon and suppresses methane cracking.

## 4. Conclusions

Synthesis gas production and oxygen exchange capacity were investigated for thermochemical redox cycling of vanadia–ceria multiphase systems with V concentrations in the range 0–100%. The materials were synthesized using a facile method involving combustion of liquid phase Ce and V precursors. Improved structural stability was achieved in mixed vanadia–ceria systems as compared to pure CeO_2_ and pure V_2_O_5_. A phase transformation of CeVO_4_ to CeVO_3_ accompanied by the formation of other segregated phases such as VO_2_ and V_2_O_3_ was observed after the thermochemical redox cycling. A mixture of CeO_2_ and CeVO_4_ with notable V concentrations showed a synergic effect in syngas yields as compared to CeO_2_ and CeVO_4_ alone. High V content facilitated carbide oxidation, which resulted in the H_2_/CO ratios as low as 2.14 due to low deposited carbon contents. The sequence of H_2_O and CO_2_ splitting reactions significantly affected the yields and rates of syngas production. Sequential H_2_O and CO_2_ splitting reactions in individual cycles improved the H_2_ purity and H_2_/CO ratio (up to 70%) as compared to H_2_O splitting alone. This study provides important information to advance the experimental investigation of metal-metal and metal-oxygen interactions in oxygen carrier material during thermochemical redox cycles.

## Data Availability

Data generated or analyzed during this study are included in this published article and its Supplementary Materials.

## Supplementary Materials

Supplementary Materials Figure SI 1: schematic of the experimental setup for thermochemical redox cycling. Figure SI 2: effect of V addition on morphology: scanning electron microscopy images of as-prepared (a) pure CeO_2_, (b) CV25, (c) CV50, (d) CV75, and (e) pure V_2_O_5_, representing the change in particle growth and size of pure CeO_2_ with the addition of V. Figure SI 3: morphological investigation of cycled V_2_O_5_–CeO_2_ systems after MPO–CDS redox cycles: scanning electron microscopy images of after MPO–CDS cycled (a) pure CeO_2_, (b) CV25, (c) CV50, (d) CV75, and (e) V_2_O_5_, showing the change in morphology of the metal oxides due to high temperature sintering and chemical reactions occurring on surface/bulk. Figure SI 4: morphological investigation of after cycled CV75: transmission electron microscopy images of CV75 (a) after reduction and (b) after MPO–WS–CDS cycles, depicting drastic changes in particle size and morphology due to high temperature sintering. Figure SI 5: surface chemical analysis of after cycled samples: X-rays photoelectron spectra of reduced CV25, CV75, and V_2_O_5_ and MPO–CDS-cycled V_2_O_5_ samples, depicting the binding energy shift and change in intensities. Table SI T1: operating parameters for ICP-OES measurements. Table SI T2: phase percentages present in as-prepared vanadia–ceria systems, quantified by the Rietveld refinement technique on XRD patterns.
