# Supplementary material for: Concentration-Dependent Solar Thermochemical CO2/H2O Splitting Performance by Vanadia–Ceria Multiphase Metal Oxide Systems
Source: Research (Wash D C). 2020 Jan 29;2020:3049534. doi: 10.34133/2020/3049534 (PMC7008271; doi:10.34133/2020/3049534)
Supplement: Supplementary Materials — Figure SI 1: schematic of the experimental setup for thermochemical redox cycling. Figure SI 2: effect of V addition on morphology: scanning electron microscopy images of as-prepared (a) pure CeO2, (b) CV25, (c) CV50, (d) CV75, and (e) pure V2O5, representing the change in particle growth and size of pure CeO2 with the addition of V. Figure SI 3: morphological investigation of cycled V2O5–CeO2 systems after MPO–CDS redox cycles: scanning electron microscopy images of after MPO–CDS cycled (a) pure CeO2, (b) CV25, (c) CV50, (d) CV75, and (e) V2O5, showing the change in morphology of the metal oxides due to high temperature sintering and chemical reactions occurring on surface/bulk. Figure SI 4: morphological investigation of after cycled CV75: transmission electron microscopy images of CV75 (a) after reduction and (b) after MPO–WS–CDS cycles, depicting drastic changes in particle size and morphology due to high temperature sintering. Figure SI 5: surface chemical analysis of after cycled samples: X-rays photoelectron spectra of reduced CV25, CV75, and V2O5 and MPO–CDS-cycled V2O5 samples, depicting the binding energy shift and change in intensities. Table SI T1: operating parameters for ICP-OES measurements. Table SI T2: phase percentages present in as-prepared vanadia–ceria systems, quantified by the Rietveld refinement technique on XRD patterns. [file 3049534.f1.docx]

**Concentration-Dependent Solar Thermochemical CO_2_/H_2_O Splitting Performance by Vanadia–Ceria Multi-Phase Metal Oxide Systems**

Asim Riaz^a^, Muhammad Umair Ali^b^, T. Gabriel Enge^c^, Takuya Tsuzuki ^a^, Adrian Lowe^a,*^, and Wojciech Lipiński^a,**^

^a^ Research School of Electrical, Energy and Materials Engineering, The Australian National University, Canberra, ACT 2601, Australia.

^b^ Department of Materials Science and Engineering, College of Engineering, Peking University, Beijing 100871, China

^c^ Research School of Earth Sciences, The Australian National University, Canberra, ACT 2601, Australia.

^*^ Corresponding author. *E-mail*: [adrian.lowe@anu.edu.au](mailto:adrian.lowe@anu.edu.au); *Tel*: +61 2 612 54881.

^**^ Corresponding author. *E-mail*: [wojciech.lipinski@anu.edu.au](mailto:wojciech.lipinski@anu.edu.au); *Tel*: +61 2 612 57896


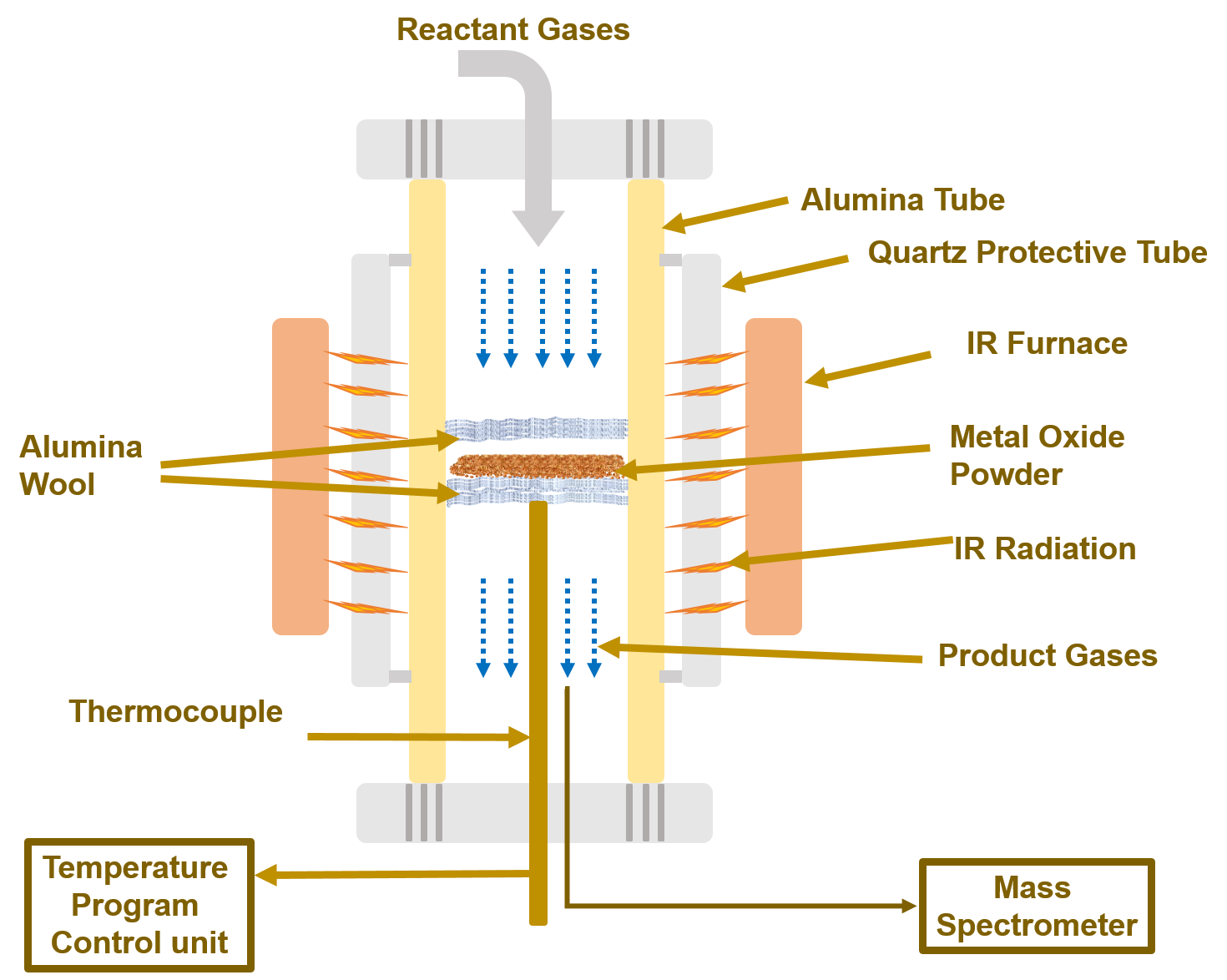


**Figure SI 1**. **Schematic of the experimental setup for thermochemical redox cycling.**

**
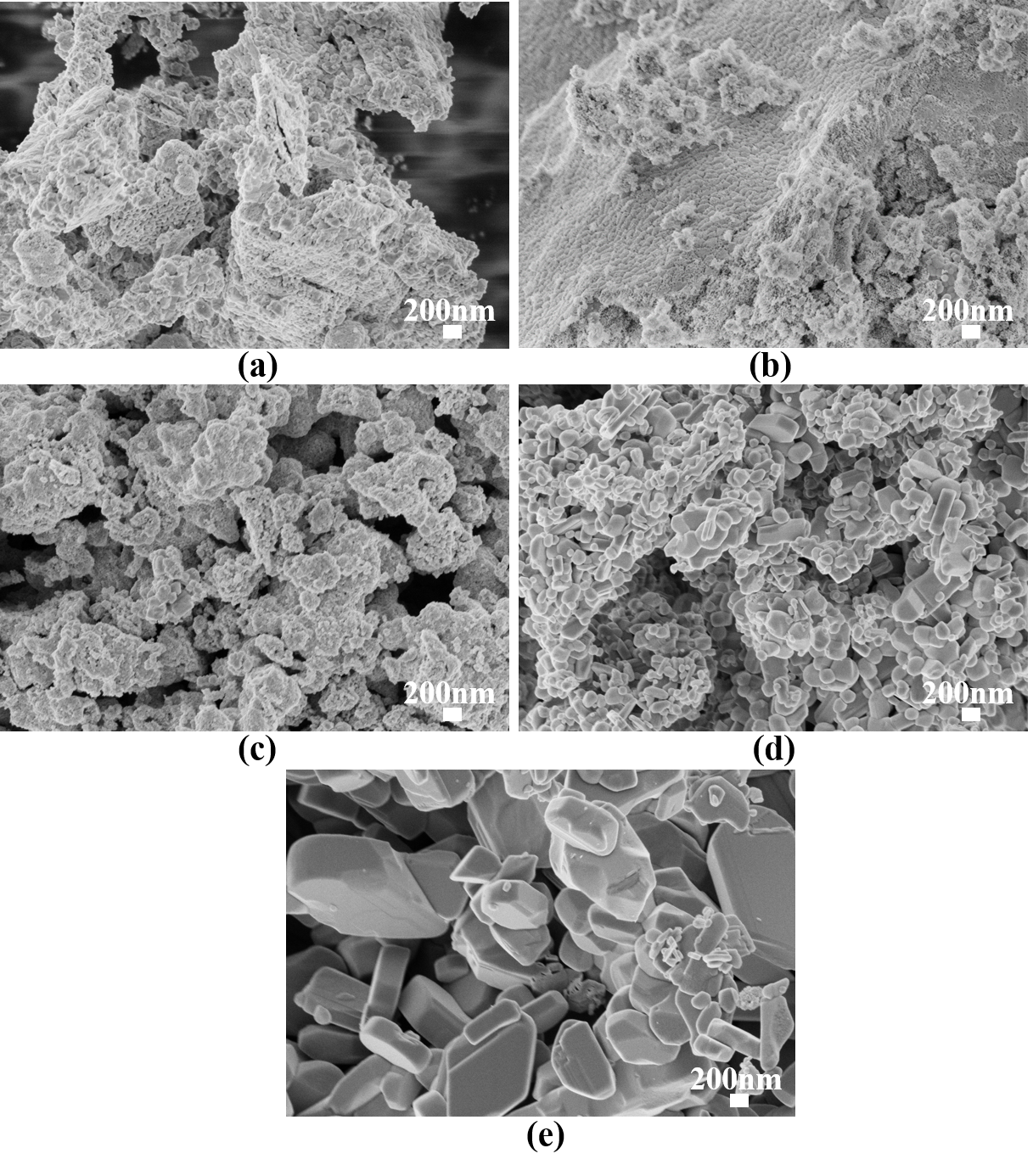
**

**Figure SI 2. Effect of V addition on morphology:** Scanning electron microscopy images of as-prepared **(a)** pure CeO_2_, **(b)** CV25, **(c)** CV50, **(d)** CV75, and **(e)** pure V_2_O_5_, representing the change in particle growth and size of pure CeO_2_ with the addition of V.


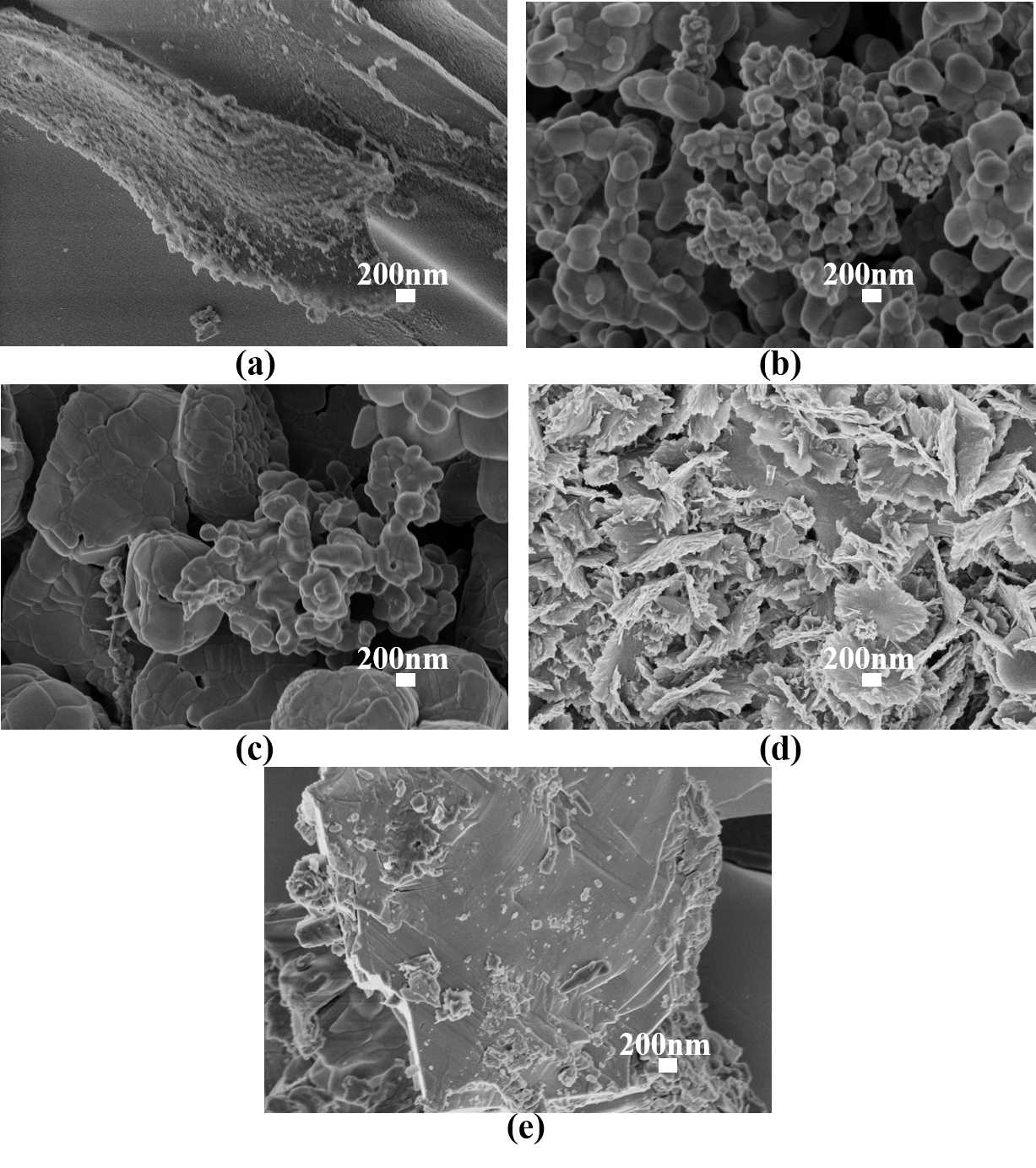


**Figure SI 3. Morphological investigation of cycled V_2_O_5_–CeO_2_ systems after MPO–CDS redox cycles:** Scanning electron microscopy images of after MPO–CDS cycled **(a)** Pure CeO_2_, **(b)** CV25, **(c)** CV50, **(d)** CV75 and **(e)** V_2_O_5_, showing the change in morphology of the metal oxides due to high temperature sintering and chemical reactions occurring on surface/bulk.


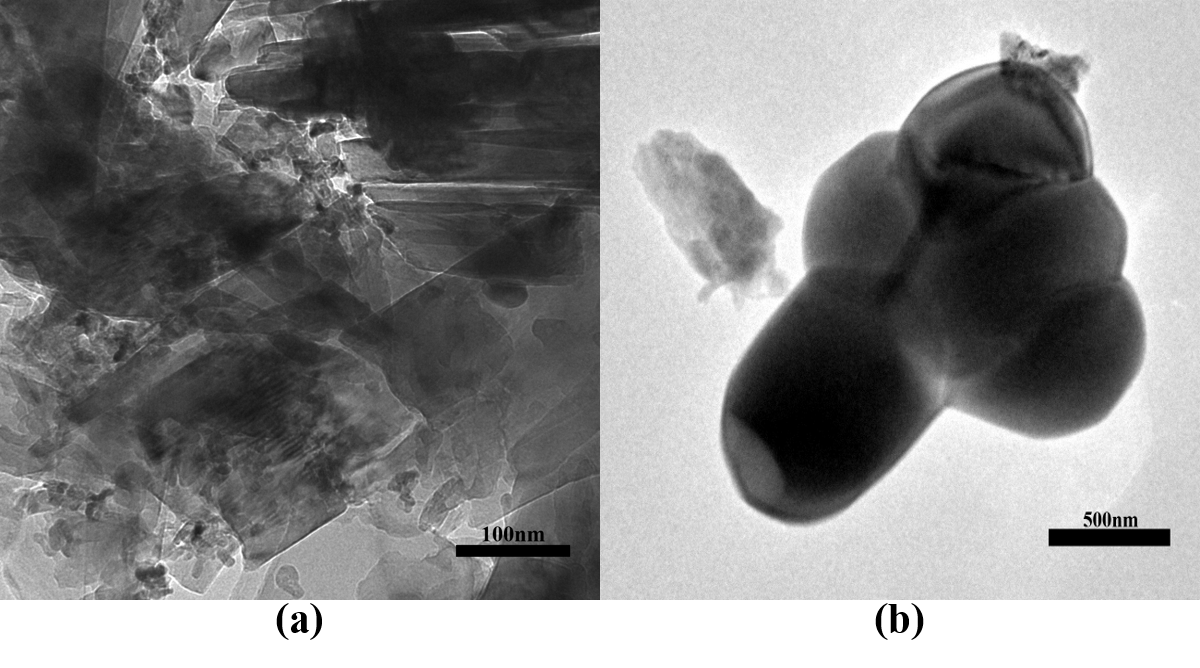


**Figure SI 4. Morphological investigation of after cycled CV75:** Transmission electron microscopy images of CV75 (a) after reduction and (b) after MPO–WS–CDS cycles, depicting drastic changes in particle size and morphology due to high temperature sintering.





**Figure SI 5. Surface chemical analysis of after cycled samples:** X-Rays photoelectron spectra of reduced CV25, CV75 and V_2_O_5_ and MPO–CDS-cycled V_2_O_5_ samples, depicting the binding energy shift and change in intensities.

**Table SI T1. Operating parameters for ICP-OES measurements.**

| Parameter | Value |
| --- | --- |
| Read time | 20 s |
| Replicates | 3 |
| Sample uptake delay | 25 s |
| Rinse time | 30 × 2 s |
| Stabilization time | 10 s |
| Pump speed | 12 rpm |
| Fast pump | ON |
| RF power | 1.5 kW |
| Aux flow | 1.0 L min^-1^ |
| Plasma flow | 12.0 L min^-1^ |
| Nebulizer flow | 0.7 L min^-1^ |
| Viewing mode | SVDV |
| Viewing height | 5 mm |
| Background correction | FACT |
| Number of pixels | 2 |
| Analytes | Ce (413.765 nm, 418.659 nm, 446.021 nm); V (290.881 nm, 292.401 nm, 309.310 nm) |
| Drift correction | C (193.027 nm); Ar (565.070 nm, 645.918 nm, 675.283 nm, 699.217 nm, 704.096 nm) |

**Table SI T2. Phase percentages present in as-prepared vanadia–ceria systems, quantified by the Rietvelt refinement technique on XRD patterns.**

| Sample | Phase composition (%) | | | | |
| --- | --- | --- | --- | --- | --- |
|  | **CeO_2_** | **CeVO_4_** | **V_2_O_3_** | **CeVO_3_** | **V_2_O_5_** |
| CeO_2_ | 100 | 0 | 0 | 0 | 0 |
| 25CV | 73.7 | 26.3 | 0 | 0 | 0 |
| 50CV | 17.8 | 76 | 6.2 | 0 | 0 |
| 75CV | 4.2 | 16.9 | 46.1 | 0.8 | 0 |
| V_2_O_5_ | 0 | 0 | 0 | 0 | 100 |
